# Impact of mental health, fear, and social support on quality of life among patients with severe mental illness during the COVID-19 pandemic: a questionnaire survey study

**DOI:** 10.3389/fpsyt.2025.1633781

**Published:** 2025-11-28

**Authors:** Sun Ju Kim

**Affiliations:** Hemodialysis Department, Chungnam National University Sejong Hospital, Sejong, Republic of Korea

**Keywords:** forensic psychiatry, mental illness, quality of life, COVID-19, anxiety, stress

## Abstract

**Purpose:**

This study aimed to assess levels of anxiety, depression, stress, fear, social support, and QoL among patients diagnosed with schizophrenia, schizoaffective disorder, bipolar disorder, or major depressive disorder receiving treatment at a national forensic psychiatric hospital during the COVID-19 pandemic.

**Methods:**

A cross-sectional survey was conducted in 2021 using a structured self-report questionnaire. Participants provided informed consent, and institutional ethical approval was obtained. Data were analyzed to examine associations among anxiety, depression, stress, fear, social support, and QoL outcomes.

**Results:**

Among the participants, 13.2% reported moderate-to-severe anxiety and 22.1% showed moderate-to-severe depressive symptoms. The average stress score was 15.63 ± 5.43, and the mean fear score was 14.13 ± 5.71, with 15.4% scoring above the clinical threshold. Perceived social support was moderate, with mean scores from healthcare providers (18.72 ± 6.43), family (18.79 ± 7.97), and friends (16.26 ± 7.46).

**Conclusion:**

The findings highlight the compounded psychological burden experienced by institutionalized patients with SMIs during a pandemic. These results underscore the need for targeted nursing interventions and psychosocial support strategies within forensic psychiatric settings to improve QoL and mental well-being during public health emergencies.

## Background

Severe mental illnesses (SMIs) refer to mental, behavioral, or affective disorders causing significant functional disruptions that limit more than one of the activities of daily living (1). In this study, SMIs refer to schizophrenia, schizoaffective disorder (SD), bipolar affective disorder (BD), and major depressive disorder (MDD).

Although the prevalence of mental illness varies depending on the type of the disorder, the main reasons for hospitalization are particularly schizophrenia and mood disorder (2). Schizophrenia is a major mental disorder that requires continuous management and supervision because its course and prognosis vary widely after onset. This chronic disease is prevalent in approximately 1% of the population, regardless of culture and ethnicity (3, 4).

Many experts reported that the recent COVID-19 pandemic may have harmed individuals who were previously diagnosed with mental disorders (5), especially those with SMIs such as schizophrenia (6) and bipolar disorder (7).

For example, a study found that individuals with SMIs may have a higher risk of the recurrence of COVID-19 due to higher levels of stress (8), and preventive strategies such as social distancing and isolation can make them more vulnerable to loneliness, thereby exacerbating their symptoms (9).

An empirical study reported that people with affective disorders (e.g., bipolar disorder or depression), compared with those without, experienced increased levels of depression, anxiety, and stress during the COVID-19 pandemic (10). Compared with the control group involving psychiatrically healthy individuals, patients with SMIs (e.g., bipolar disorder or schizophrenia) experienced more pronounced symptoms of depression, anxiety, and stress (11).

A study conducted in India found that 30% of patients with SMIs experienced a relapse during the COVID-19 pandemic (12). Interestingly, compared with patients with bipolar disorder, those with affective disorder had higher stress levels due to the fear of COVID-19 infection (13). Meanwhile, when patients with mental illnesses were provided with social support, positive impacts such as lower stress levels and recurrence rates, and improved quality of life (QoL) were observed (14).

The current study is grounded in the stress-vulnerability model, which posits that individuals with severe mental illness possess an underlying biological or psychological vulnerability that interacts with environmental stressors to determine mental health outcomes (15). During the COVID-19 pandemic, restrictive institutional measures, fear of infection, and disrupted social contact served as significant stressors that could exacerbate psychological distress among this population. Conversely, social support functions as a protective factor that can buffer the negative impact of stress and promote adaptive coping. Based on this model, the present study hypothesizes that heightened fear and psychological distress would be associated with poorer quality of life, whereas stronger social support would mitigate these effects. Integrating this theoretical framework allows for a more comprehensive interpretation of the interrelationships among mental health, fear, social support, and quality of life in institutionalized patients with severe mental illness.

COVID-19 is a disease that affects almost every country. Recent research has increasingly examined the psychosocial and quality-of-life outcomes of individuals with severe mental illness (SMI) during the COVID-19 pandemic. For instance, *Tripoli* et al. (2024) (16) investigated lifestyle patterns and quality of life among psychiatric patients during the pandemic and found that social isolation and disrupted routines were significant predictors of lower well-being. Similarly, *van Rijn* et al. (2025) (17) reported that adults with SMI in Dutch longitudinal cohorts experienced notable declines in psychosocial functioning and life satisfaction during the early pandemic phase, although some recovery patterns emerged over time. These findings underscore that SMI populations are disproportionately affected by pandemic-related restrictions. The current study extends this line of research by focusing on institutionalized forensic patients—a uniquely vulnerable subgroup subject to prolonged confinement and limited family contact—thereby contributing new evidence on how mental health, fear, and perceived social support interact to shape quality of life in this context. Considering the scenario presented above, this study aimed to assess anxiety, depression, stress, fear, social support, and QoL among patients with schizophrenia, SD, BD, and MDD in the National Forensic Psychiatric Hospital throughout the COVID-19 pandemic. This study also aimed to identify factors influencing the QoL of individuals with mental illness to provide foundational data for developing a QoL enhancement program for patients with SMIs in preparation for future pandemics. Therefore, this study aimed to examine the QoL of patients with SMIs in a forensic psychiatric hospital and identify the factors influencing it. The study’s specific objectives were as follows:

1. To examine participants’ general characteristics, as well as their levels of anxiety, depression, stress, fear, social support, and QoL.
2. To analyze the correlations between anxiety, depression, stress, fear, social support, and QoL among participants.
3. To identify the factors (depression, anxiety, stress, fear, and social support) influencing participants’ QoL.

The following hypotheses were proposed:

1. As the participants have lower levels of anxiety, depression, stress, and fear, they will have an improved QoL.
2. As the participants have increased social support, they will have an improved QoL.

## Methods

### Data collection

A self-reported questionnaire survey was conducted from November 2021 to December 2021.

### Participants

Patients undergoing treatment in the National Forensic Hospital were selected as participants in this study. The inclusion criteria were male and female adults aged 18 years or above who were diagnosed with an SMI (schizophrenia, SD, BD, and MDD) and could read questions and express their opinions. This study excluded patients with brain damage, intellectual disabilities, and dementia.

Participants were recruited from a National Forensic Psychiatric Hospital in South Korea. All were inpatients receiving mandatory treatment under court orders following criminal proceedings, consistent with the criteria of the Mental Health Act. Individuals found not guilty by reason of insanity or deemed criminally irresponsible due to psychiatric disorders were included. This legal and clinical status differentiates the sample from general psychiatric inpatients and must be considered when interpreting external validity.

Ethical approval was obtained from the institutional review board, and written informed consent was secured from all participants. The final sample comprised 136 patients (89% male), aged 20–65 years, diagnosed with schizophrenia, schizoaffective disorder, bipolar disorder, or major depressive disorder.

After obtaining approval from the Institutional Review Board, we recruited researchers, sought cooperation from each ward in the institution, and posted a recruitment notice. After the research assistant explained the purpose and content of the study to the participants who voluntarily expressed their willingness to participate, we directly obtained their consent and proceeded with the study. We clearly stated and explained that there would be no disadvantages resulting from the termination of treatment supervision and discharge.

The appropriate sample size was calculated using G*Power 3.1.9.2 (18). With a significance level of.05, statistical power (1-β) of.90, medium effect size of 0.15 for regression analysis, and five independent variables, the required sample size was determined to be 116. Allowing for a 10% dropout rate, the final sample size was set at 129.

**Figure 1:**
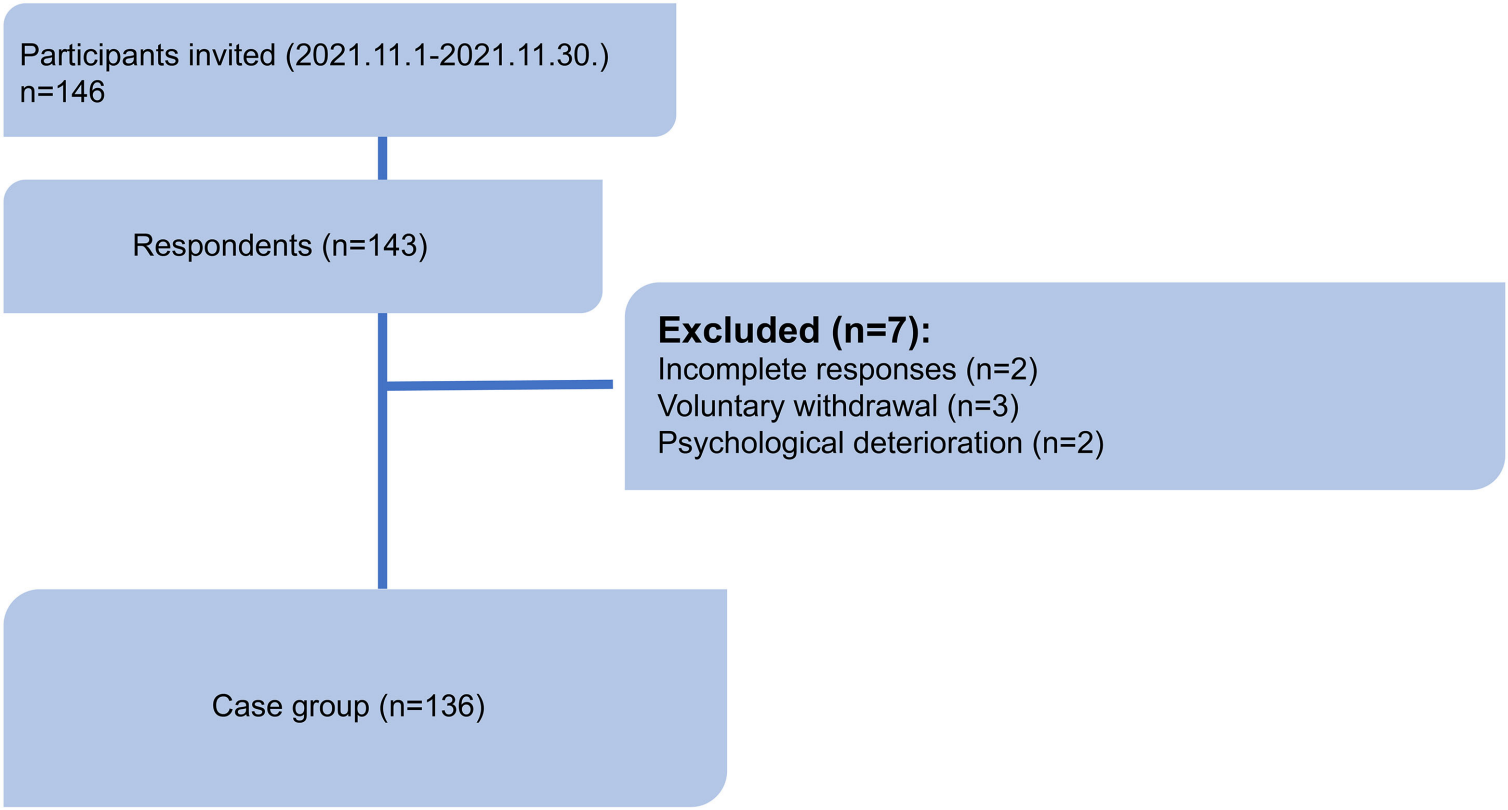
Study flow diagram.

A total of 146 individuals were invited to participate in the study, and 143 responded (response rate: 97.9%). Among these, seven participants were excluded due to incomplete responses (n=2), voluntary withdrawal (n=3) and psychological deterioration (n=2). The final analytical sample consisted of 136 participants. The study flow diagram is presented in **Figure 1**.

The overall proportion of missing data was less than 10%, and missing data were distributed randomly across variables. Considering the low proportion of missing data, no imputation methods were applied, and complete case analysis was conducted.

### Variables

#### Anxiety

The Korean version of the Beck Anxiety Inventory (BAI), a tool for analyzing the frequency of anxiety within a period of more than a week, was used in this study (19, 20). The BAI is a self-reported measure comprising 21 items, with responses measured using a Likert scale ranging from 0 to 3. This tool is used to measure the level of anxiety a person has due to symptoms described in each question, within the past week. The Cronbach’s α of the BAI was.92 in this study.

#### Depression

This study used the Beck Depression Inventory (BDI), originally developed by Beck et al. (21) and Lee (22). The BDI comprises 21 items rated on a 4-point Likert scale, with scores ranging from 0 to 63. Scores of 0–9 indicate normal, 10–15 indicate mild, 16–23 indicate moderate, and 24–63 indicate severe depression, with higher scores reflecting greater levels of depression. When this tool was initially developed, the Cronbach’s α was.86, and it was.90 in this study.

#### Perceived stress scale

The PSS, developed by Cohen et al. (23) is a 14-item questionnaire designed for assessing stressful experiences of participants during the past month. The Korean version of the scale with 10 items modified by Cohen (24) and translated by Lee (25) was used in this study. The range of possible scores is 0 to 40, with responses measured using a 5-point Likert scale and higher scores indicating greater levels of stress. The Cronbach’s α was.84 at development,.78 after modification, and.84 in this study.

#### The fear of COVID-19 scale

The FCV-19S, developed by Ahorsu et al. (26) and translated into Korean by Han et al. (27), was used in this study. The responses to the individual items are rated on a 5-point Likert scale, with total scores ranging from 7 to 35; higher scores indicate greater fear of COVID-19. The tool’s Cronbach’s α was.82 at development, test–retest reliability was 0.72, and Cronbach’s α in this study was.86.

#### Multidimensional scale of perceived social support

The MSPSS, a 12-item scale developed by Zimet et al. (28) and translated into Korean by Park et al. (29), was used in this study to measure the perceived social support from family, friends, and significant others, with “significant other” adapted to indicate healthcare provider support. Responses were rated on a 7-point Likert scale, with higher scores reflecting greater social support. The tool’s Cronbach’s α was.91 at development and.94 in this study.

#### EuroQoL (EQ-5D-5L)

The EQ-5D-5L, a widely used tool for assessing health-related QoL, consists of five items: Mobility, Self-Care, Usual Activities, Pain/Discomfort, and Anxiety/Depression. This study utilized the Korean version of the EQ-5D, developed by Nam et al. (30) based on the EuroQoL-5D (EQ-5D) from the EuroQoL Group. Each item has five response options: no problems (level 1), slight problems (level 2), moderate problems (level 3), severe problems (level 4), and extreme problems (level 5). The EQ-5D Index ranges from −0.0171 to 1, with lower values indicating poorer health, and its Cronbach’s α was.76. Additionally, the EuroQoL Visual Analog Scale (EQ-VAS), a visual analog scale, rates health from 0 to 100, with 100 being the best imaginable health state and 0 being the worst, indicating both health outcome order and preference degree.

### Data analysis

Data were analyzed using SPSS version 22.0. Descriptive statistics (frequency, percentage, mean, and standard deviation) were used to summarize participants’ demographic and clinical characteristics, including anxiety, depression, perceived stress, fear of COVID-19, social support, and quality of life (QoL). Cronbach’s α was calculated to assess the internal consistency of all measurement instruments.

Independent t-tests and one-way ANOVA were performed to examine differences in QoL according to general characteristics. Pearson correlation analyses were used to explore associations among psychological variables and QoL. To identify predictors of QoL, hierarchical multiple regression analyses were conducted. Variables were entered in three sequential steps: (1) demographic variables, (2) psychological distress variables (anxiety, depression, perceived stress), and (3) social factors (fear of COVID-19 and perceived social support). Multicollinearity was assessed using variance inflation factors (VIF < 2.0), and model independence was verified using the Durbin–Watson statistic.

### Ethical considerations

After receiving approval from the Institutional Review Board of the National Forensic Psychiatric Hospital (1-219577-AB-N-01-202110-HR-004-01) on Oct 13, 2021 (Approval date), participants were informed about the purpose and details of this study, assured of the confidentiality and anonymity of their data, and assured of the data’s usage for academic purposes only. Additionally, they were informed of the voluntary nature of their participation and their scope of withdrawal at any time. We also explained that there would be no disadvantages in case of dropout and obtained written consent from each participant before collecting data. We explained that there would be no disadvantages associated with conditional release or discharge due to participation refusal. This study was conducted in accordance with the principles outlined in the Declaration of Helsinki and adhered to applicable institutional ethical guidelines. Clinical trial number: not applicable.

## Results

### Demographic characteristics

The average age of the 136 participants was 45.61 years, with those aged 50 years and above accounting for the highest proportion at 41.2%. Of the total, 89.0% were male, 71.3% were unmarried, 59.6% were middle/high school graduates, 69.1% were those with no children, 51.5% were those with low socioeconomic status, and 68.4% were those with a religion. The majority of the participants (95.6%) received COVID-19 management education, 54.9% responded that the effect of COVID-19 education is above average, and most of them answered that they understand about COVID-19. A total of 18.4% experienced isolation due to COVID-19, and 95.6% were vaccinated against COVID-19; 55.9% expressed a fear of infection within their family, 92.0% responded that they receive support from nurses, 96.3% responded that they are confident in coping with COVID-19, and 93.4% answered that their fear of infection decreased after vaccination (see **Table 1**).

**Table 1:** Demographic characteristics, mental health, perceived social support, and quality of life (N = 136).

	Variables	Categories	n (%) or M ± SD			
Demographic factors	Gender	Men	121	89		
		Women	15	11		
	Age (in years)	<30	11	8.1		
		30–39	21	15.4		
		40–49	48	35.3		
		≥50	56	41.2		
			45.61	± 9.65		
	Marital status	Unmarried	97	71.3		
		Married	39	28.7		
	Educational level	Elementary	7	5.1		
		Middle/High	81	59.6		
		Bachelor’s degree	43	31.6		
		Postgraduate degree	5	3.7		
	Presence of children	Yes	32	23.5		
		No	94	69.1		
		Not applicable	10	7.4		
	Socioeconomic status	Low	70	51.5		
		Middle	61	44.9		
		High	5	3.7		
	Religion	Yes	93	68.4		
		No	43	31.6		
	Education on COVID-19 management	Yes	130	95.6		
		No	6	4.4		
	Effect of COVID-19 education	Disagree	20	12.3		
		Moderate	21	13		
		Agree	19	11.7		
		Strongly agree	49	30.2		
		Not applicable	14	8.6		
	Understanding of COVID-19	Disagree	3	2.2		
		Moderate	62	45.6		
		Agree	50	36.8		
		Strongly agree	21	15.4		
	COVID-19 isolation experience	Yes	25	18.4		
		No	111	81.6		
	COVID-19 vaccination	Yes	130	95.6		
		No	6	4.4		
	Fear of infection in the family	Yes	76	55.9		
		No	60	44.1		
	Support of nurse	Strongly disagree	4	2.9		
		Disagree	7	5.1		
		Moderate	58	42.6		
		Agree	51	37.5		
		Strongly agree	16	11.8		
	COVID-19 coping confidence	Disagree	5	3.7		
		Moderate	63	46.3		
		Agree	51	37.5		
		Strongly agree	17	12.5		
	Reduction of infection	Strongly disagree	5	3.7		
	Anxiety after COVID-19 vaccination					
		Disagree	4	2.9		
		Moderate	33	24.3		
		Agree	55	40.4		
		Strongly agree	37	27.2		
Psychological factors	Variables	Categories	n	(%)	M	± SD
	BAI	Normal (0-7)	90	66.2%	2.53	2.38
		Mild (8-15)	28	20.6%	10.96	2.46
		Moderate (16–25)	14	10.3%	21.07	2.40
		Severe (26–63)	4	2.9%	30.75	1.26
	BDI	Normal (0–13)	84	61.8%	5.55	3.71
		Mild (14–19)	22	16.2%	15.95	1.65
		Moderate (20–28)	22	16.2%	23.27	2.31
		Severe (29–63)	8	5.9%	35.75	9.47
	PSS	Normal (0–13)			15.63	5.43
		Mild (14–16)				
		Moderate (17–18)				
		Severe (>19)				
FCV-19S	FCV-19S total score	≤20.00	115	84.6%	12.40	4.19
		21.00–30.00	21	15.4%	23.57	3.01
MSPSS	Significant other support				18.72	6.43
	Family support				18.79	7.97
	Friend support				16.26	7.46
EQ-5D-5L					0.83	0.11
EQ-VAS					72.50	15.75

BAI, Beck Anxiety Inventory; BDI, Beck Depression Inventory; PSS, Perceived Stress Scale; FCV-19S, Fear of COVID-19 Scale; MSPSS, Multidimensional Scale of Perceived Social Support; EQ-5D-5L, EuroQoL; EQ-VAS, EuroQoL Visual Analog Scale.

### Depression, anxiety, stress, fear, social support, and QoL

The mean anxiety score measured by the Beck Anxiety Inventory (BAI) was 7.01 ± 7.70), which falls below the clinical threshold of 16 points, indicating non-clinical levels of anxiety in this sample. However, 13.2% of the participants exhibited moderate-to-severe anxiety. Similarly, the mean depression score assessed using the Beck Depression Inventory (BDI) was 11.88 ± 9.85, which is below the clinical threshold of 20 points, although 22.1% of the participants showed moderate-to-severe levels of depression. The mean perceived stress score was 15.63 ± 5.43. The mean score of fear was 14.13 ± 5.71, with 15.4% having a score of 21 or above. The mean score of social support from healthcare providers was 18.72 ± 6.43, the mean score of family support was 18.79 ± 7.97, and that from a friend/s was 16.26 ± 7.46. Among the domains of QoL, on average, the EQ-5D5L score was 0.83 ± 0.11 and the EQ-VAS score was 72.50 ± 15.75 (**Table 1**).

### Differences in anxiety, depression, stress, fear, social support, and QoL based on general characteristics

Gender and the presence of children showed statistically significant differences in the relationship between general characteristics and QoL (EQ-5D Index). The QoL was higher among men (t=2.66, p=.012) and among participants with children (t=4.54, p=.018) compared with women and participants without children. Furthermore, significant differences were noted regarding marital status, presence of children, presence of fear of infection from family, and COVID-19 coping confidence in the relationship between general characteristics and the EQ-VAS score. The subjective health state on the day of questionnaire completion was higher among participants who were married (t=−2.17, p=.032), those with children (t=5.39, p=.006), and those who had no fear of infection from family (F = 2.77, p=.044) (**Table 2**).

**Table 2:** Differences in quality of life based on demographic characteristics (N = 136).

Variables	Categories	EQ-5D Index				EQ-5D VAS†			
		M	± SD	t or F	p	M	± SD	t or F	p
Gender	Men	0.82	0.11	−2.97	.01	72.69	15.23	0.41	.68
	Women	0.87	0.04			70.93	20.01		
Age (in years)	<30	0.80	0.14	1.56	.20	73.18	12.54	2.09	.11
	30–39	0.87	0.05			80.14	16.60		
	40–49	0.82	0.11			70.52	17.37		
	≥50	0.82	0.11			71.20	13.94		
Marital status	Unmarried	0.83	0.10	0.55	.58	74.33	15.60	−2.17	.03
	Married	0.84	0.11			67.95	15.37		
Educational level	Elementary	0.84	0.07	0.05	.99	68.43	19.03	1.02	.39
	Middle/High	0.83	0.11			71.06	15.29		
	Bachelor’s degree	0.83	0.11			75.81	16.53		
	Postgraduate degree	0.82	0.15			73.00	9.75		
Socioeconomic status	Low	0.82	0.11	1.09	.37	69.81	16.11	2.64	.08
	Middle	0.84	0.10			74.80	15.06		
	High	0.77	0.18			82.00	13.04		
Presence of children	Yes	0.82	0.13	7.34	<.001	67.19	15.45	5.39	.01
	No	0.83	0.10			72.97	15.42		
	Not applicable	0.88	0.03			85.10	12.73		
Religion	Yes	0.82	0.11	−1.04	.30	71.96	15.08	−0.59	.56
	No	0.84	0.09			73.67	17.24		
Education on COVID-19 management	Yes	0.83	0.11	−0.76	.45	72.45	16.07	−0.16	.87
	No	0.86	0.04			73.50	5.50		
Effect of COVID-19 education	Disagree	0.79	0.10	0.99	.42	68.80	21.61	0.18	.95
	Moderate	0.81	0.13			72.40	13.88		
	Agree	0.84	0.09			73.31	17.32		
	Strongly agree	0.84	0.10			70.84	16.82		
	Not applicable	0.86	0.03			75.25	6.18		
Understanding of COVID-19	Disagree	0.84	0.11	0.46	.71	76.00	22.87	0.48	.70
	Moderate	0.82	0.10			73.05	14.37		
	Agree	0.82	0.12			73.16	16.71		
	Strongly agree	0.85	0.08			68.81	17.02		
COVID-19 isolation experience	Yes	0.80	0.13	−1.42	.17	30.29	5.23		
	No	0.84	0.10			32.11	5.73		
COVID-19 Vaccination	Yes	0.83	0.10	1.46	.20	72.36	15.92	−0.48	.64
	No	0.72	0.18			75.50	11.88		
Fear of infection in the family	Yes	0.82	0.11	0.84	.36	69.05	16.32	8.73	<.001
	No	0.84	0.10			76.87	13.94		
Support of nurse	Strongly disagree	0.78	0.12	2.24	.07	69.75	32.10	0.32	.86
	Disagree	0.74	0.15			70.57	15.30		
	Moderate	0.82	0.11			72.10	14.90		
	Agree	0.85	0.09			74.35	15.57		
	Strongly agree	0.84	0.10			69.56	15.97		
COVID-19 coping confidence	Disagree	0.86	0.06	0.24	.87	78.60	11.82	2.77	.04
	Moderate	0.83	0.10			70.22	15.05		
	Agree	0.83	0.10			76.67	15.37		
	Strongly agree	0.82	0.14			66.65	17.83		
Reduction of infection anxiety after COVID-19 vaccination	Strongly disagree	0.83	0.11	0.37	.86	69.00	17.46	0.25	.94
	Disagree	0.72	0.25			69.75	7.76		
	Moderate	0.82	0.09			73.27	14.22		
	Agree	0.84	0.08			73.51	14.92		
	Strongly agree	0.84	0.10			70.81	18.74		

The symbol † indicates the EQ-VAS score.

### Correlations between depression, anxiety, stress, fear, social support, and QoL

Significant correlations were found between anxiety, depression, stress, social support (healthcare provider, family, and friend support), and QoL (**Table 3**). The QoL (EQ-5D Index) had negative correlations with anxiety (r=−0.55, p<.001), depression (r=−0.36, p<.001), and stress (r=−0.27, p=.002), and it had positive correlations with social support (r=0.19, p=.028) and support from friend/s (r=0.21, p=.016). Essentially, lower levels of anxiety, depression, and stress and higher social support indicated an improved QoL.

**Table 3:** Correlations between anxiety, depression, stress, fear, perceived social support, and quality of life (N = 136).

	BAI	BDI	PSS	FEAR	Significant others support	Family support	Friend support	MSPSS	EQVAS	Quality of life
BAI	1									
BDI	.504^**^	1								
PSS	.472^**^	.420^**^	1							
FEAR	.182^*^	.200^*^	.182^*^	1						
Significant others support	−.178^*^	−.190^*^	−.173^*^	−.044	1					
Family support	−.188^*^	−.201^*^	−.340^**^	−.1	.621^**^	1				
Friend support	−.166	−.221^**^	−.359^**^	−.115	.560^**^	.697^**^	1			
MSPSS	−.204^*^	−.236^**^	−.342^**^	−.102	.819^**^	.903^**^	.875^**^	1		
EQ-VAS	−.240^**^	−.296^**^	−.281^**^	−.296^**^	.125	.235^**^	.345^**^	.277^**^	1	
Quality of life	−.553^**^	−.357^**^	−.269^**^	−.136	.158	.127	.207^*^	.188^*^	.375^**^	1

*p<.05, †Visual Analog Scale.

BAI, Beck Anxiety Inventory; BDI, Beck Depression Inventory; MSPSS, Multidimensional Scale of Perceived Social Support; PSS, Perceived Social Support; EQ-VAS, EuroQoL Visual Analog Scale.

*p<.05; **p<.01.

### Correlates of QoL

Although QoL comprises the EQ-5D Index and EQ-VAS, the latter does not reflect the culture and situation of each country, making it difficult to compare scores between studies. Therefore, the EQ-5D Index that reflects Koreans’ QoL was used (31, 32). A hierarchical multiple regression analysis was performed to examine factors influencing participants’ QoL, with the results as follows (**Table 4**).

**Table 4:** Multiple regression results for EQ-5D-5L (N = 136).

Variables	Model 1				Model 2				Tolerance	VIF
	B	SE	β	P	B	SE	β	P		
Constant	0.92	0.04		.00	0.91	0.05		.00		
Gender (male)	−0.05	0.03	−0.15	.09	−0.04	0.03	−0.11	.16	0.93	1.08
Presence of children (Yes)	−0.07	0.04	−0.28	.07	−0.04	0.03	−0.18	.18	0.29	3.43
Presence of children (No)	−0.05	0.04	−0.21	.17	−0.03	0.03	−0.12	.35	0.30	3.34
BAI					−0.01	0.00	−0.50	<.001	0.65	1.53
BDI					0.00	0.00	−0.09	.33	0.68	1.47
PSS					0.00	0.00	0.04	.63	0.66	1.51
Friend support					0.00	0.00	0.11	.18	0.84	1.19
Adj R^2^	0.02				0.31					
⊿R^2^					0.30					
F (p)	1.87	0.14			9.60	<.001				

BAI, Beck Anxiety Inventory; BDI, Beck Depression Inventory; PSS, Perceived Social Support; VIF, Variance Inflation Factor; EQ-5D-5L, EuroQoL.

As a result of verifying the regression analysis hypotheses, the overall model-fit was acceptable. The Durbin–Watson statistic was 2.097, indicating no autocorrelation. The variance inflation factor ranged from 1.108 to 7.748, remaining below 10, which indicates no multicollinearity.

This study used the gender of the participants and the presence of children in their lives as control variables as they showed significant effects in the difference analysis between the general/psychological characteristics and QoL. Therefore, in the first step, gender and the presence of children were regressed into the QoL, an outcome variable (Model 1), and in the second step, anxiety, depression, stress, and friend support were regressed into the QoL (Model 2).

The explanatory power of Model 1 that includes two control variables was 1.9% (F = 1.874, p=.137), with no significant control variables for QoL. Although the individual predictors did not achieve conventional levels of significance, the standardized effect sizes indicate that having children (Yes) may be relatively more influential in predicting lower QoL compared to gender or presence of children (No). These findings provide preliminary insights into the demographic factors influencing QoL among patients with severe mental illness during the COVID-19 pandemic, as measured using the EQ-5D-5L, and underscore the need for further investigation using additional psychological and social support variables. The explanatory power of Model 2 that includes psychological characteristics was 30.8% (F = 9.600, p<.001). In Model 2, anxiety among the control variables was found to affect QoL (β=−0.500, p<.001). Among the predictors, the BAI emerged as the most influential factor, demonstrating a large negative effect on QoL (B = –0.01, SE < 0.01, β = –0.50, p <.001). This finding indicates that a one standard deviation increase in anxiety is associated with a 0.50 standard deviation decrease in EQ-5D-5L scores, underscoring the clinical significance of anxiety in this population. In contrast, demographic variables, such as gender and presence of children, and other psychological variables, including the BDI, PSS, and friend support, contributed minimally to the prediction of QoL. Overall, these results highlight that while the inclusion of demographic and psychosocial variables collectively improves the explanatory power of the model, anxiety—as measured using the BAI—stands out as the most robust and clinically relevant predictor of lower EQ-5D-5L scores. This suggests that interventions aimed at reducing anxiety may have a meaningful impact on improving QoL among patients with severe mental illness during the COVID-19 pandemic.

## Discussion

In this study, 13.2% of patients exhibited moderate or higher levels of anxiety, and 22.1% showed moderate or higher levels of depression. In a previous study on outpatients with affective disorder, 26% showed a moderate or above level of anxiety, 17% showed a moderate or above level of depression, and 7% showed post-traumatic stress disorder (33). This indicates that there are differences between studies.

In a prior study (34), significantly high scores were noted in the subscales of anxiety (p=.04) and stress (p=.05). Participants diagnosed with BD reported a significant increase in suicidal ideation following the COVID-19 pandemic (p=.01).

Furthermore, the QoL score was significantly low among patients with BD (p=.02). This phenomenon was noticeable among patients who complained of economic difficulties due to the lockdown and was more pronounced among patients with more maladaptive lifestyle behaviors. Similarly, this study found that patients had moderate or higher levels of anxiety and depression probably because family visits were restricted.

In this study, patients had an average stress score of 15.63 ± 5.43, indicating they had mild stress. Yocum et al. (35) also reported that outpatients with BD were highly likely to experience stress related to infectious disease during the early stage (April 30, 2020, lockdown week 5) (p<.01). Compared with the healthy control group, individuals with BD experienced slower recovery due to disrupted daily routines and insufficient social support (35). Regarding this study’s findings, it is thought that the experience of isolation increased stress levels among some of the patients in this study.

The participants’ mean score of fear was 14.13 ± 5.71, with 15.4% scoring 21 or above. A previous study on schizophrenia, BD, and MDD, which was conducted by Chang et al. (36), reported that patients who more strongly believed in COVID-19 information from newspapers, television, and online sources were more frightened of COVID-19. Therefore, previous findings suggest that high levels of fear are associated with depression, anxiety, and stress and preventive measures for COVID-19 may negatively impact individuals with mental disorders. 15.4% of them showed fear, with a score of 21 or above, suggesting that prevention and treatment management should be carefully provided to patients who have a fear of infection.

The perceived social support scores from healthcare providers, family, and friends were 18.72 ± 6.43, 18.79 ± 7.97, and 16.26 ± 7.46, respectively. These scores were in contrast with those of a study conducted by Hofer et al. (37) in which patients with MDD and SMIs reported lower social support compared with the control group. The perception of the COVID-19 pandemic and related public health policies as distressing is believed to have affected the patients (38).

The average EQ-5D-5L score within the QoL domains was 0.82 ± 0.11, which is higher than that of a study on patients with schizophrenia (0.80) (39) and lower than a previous study (0.86) (40). A study conducted by Karantonis et al. (34) also reported that the QoL score for patients with BD was significantly low (p=.02). In this study and previous studies, the QoL of patients with SMIs was generally low. In particular, the participants of this study were considered affected by the closed environment during the COVID-19 pandemic, even though they could have requested adjustments to mobility, self-care, usual activities, and pain/discomfort within the hospital.

Studies on MDD, anxiety disorder, post-traumatic stress disorder, attention deficit hyperactivity disorder, insomnia, and others reported that increased anxiety about COVID-19, increased sleep problems (41), and fatigue (42) were related to lower QoL among individuals who were diagnosed with or suspected of having mental illnesses. This might be because of several factors such as multimorbidity, psychological symptoms arising due to COVID-19 variant infections, and prolonged restrictions on visits. Further research on factors that influence QoL according to diagnosis should be conducted.

Women’s QoL was found to be lower than that of men in this study (t=2.66, p=.012). This is similar to the results of the study conducted by Al-Shannaq et al. (43); a probable reason for this result is that women tend to be concerned about their family being infected by the virus before themselves, and this affects their psychological well-being and consequently, their QoL.

In Model 2, anxiety among the control variables was found to affect QoL. A study conducted by Li et al. (44) also reported that patients with anxiety showed lower QoL compared with those with no anxiety. Quality of life (QoL) is determined by improved social support and the interaction between mental health and physical condition (45). Anxiety can be related to cognitive function disorders (46), physical pain (47), and social function disorders (48) that in turn decrease patients’ QoL. In this study, as the participants were not allowed visits from family and friends due to preventive measures for COVID-19, the fear of infection with the Omicron variant seemed to lead to lower QoL.

The hierarchical regression model explained 30.8% of the variance in quality of life, suggesting that additional unmeasured variables may influence outcomes. Factors such as resilience, self-efficacy, and coping strategies could provide further explanatory power. Subsequent studies incorporating these constructs or testing mediating pathways may yield a more comprehensive understanding of quality-of-life determinants in this population.

### Limitations

The findings should be interpreted with caution due to the study’s single-site design and the predominance of male participants (89%). These characteristics limit generalizability to other psychiatric settings and to female populations with severe mental illness. Future multi-center studies involving diverse clinical environments and balanced gender representation are warranted to confirm the robustness of the observed associations.

As the data were collected in late 2021, psychological responses may have differed from those observed during the early phase of the pandemic. Therefore, caution is needed when comparing our findings with studies conducted at earlier stages of COVID-19.

It is important to note that the data were collected in late 2021, a period following the widespread implementation of vaccination campaigns. Consequently, participants’ fear and anxiety levels might have been lower compared with those observed during the early phases of the pandemic.

Furthermore, the cross-sectional nature of this study precludes causal inference; therefore, longitudinal investigations are needed to clarify the temporal dynamics among anxiety, social support, and quality of life.

Because this study was conducted with patients of only one hospital, the findings might not represent all patients, requiring caution in generalizing the study results. Furthermore, as this study was an observational investigation, causal relationships were not examined. The determined predictor variables explained just 30% of the total variance for each model. Therefore, further research is warranted to measure variables related to the QoL of other patients and to investigate and propose measures for improving their QoL.

## Conclusions

From a clinical standpoint, our results underscore the need for routine anxiety screening and tailored psychological interventions in forensic psychiatric hospitals. Anxiety management programs, stress-coping training, and social-support enhancement strategies—such as structured family contact and staff-mediated social activities—should be prioritized. At a policy level, the findings highlight the importance of pandemic preparedness frameworks that safeguard the mental well-being of institutionalized psychiatric populations through proactive resource allocation and communication policies.

This study found that, patients with SMIs experienced lower QoL; anxiety was an influencing factor in this context. Further research using the EQ-5D-5L scale, focused on factors influencing the QoL of patients with SMIs and various nursing techniques, is necessary to obtain valuable data for enhancing their QoL.

Future research should employ longitudinal and experimental designs to examine the causal mechanisms linking anxiety and social support to quality of life. Including non-institutionalized control groups or pre-pandemic baseline data would enable clearer differentiation of COVID-19–specific effects from underlying disease-related factors.

## Data Availability

The raw data supporting the conclusions of this article will be made available by the authors, without undue reservation.
